# The Effects of Employees’ Perceived Intrinsic Motivation on Knowledge Sharing and Creative Self-Efficacy

**DOI:** 10.3389/fpsyg.2021.762994

**Published:** 2022-01-13

**Authors:** Yu Sun, Jon-Chao Hong, Jian-Hong Ye

**Affiliations:** ^1^Department of Industrial Education, National Taiwan Normal University, Taipei, Taiwan; ^2^Institute for Research Excellence in Learning Sciences, National Taiwan Normal University, Taipei, Taiwan; ^3^Faculty of Education, Beijing Normal University, Beijing, China; ^4^Dhurakij Pundit University, Bangkok, Thailand

**Keywords:** creativity self-efficacy, intelligent transportation, intrinsic motivation, knowledge sharing, system thinking

## Abstract

Knowledge sharing is the major driving force to maintain enterprises’ competitiveness. This study extends the current knowledge-sharing research by considering knowledge sharing as comprising four types: automatic response, rational reflection, ridiculed reflection, and deprived reflection, based on Kahneman’s (2011) types of system thinking. Drawing on the motivation-action-outcome model, this study explored how individuals’ intrinsic motivation can guide the action of knowledge sharing and reflect the outcome of creative self-efficacy in intelligent transportation jobs. By snowball sampling in intelligent transportation companies, a total of 232 effective questionnaires were collected, and confirmatory factor analysis with structural equation modeling was performed. The research results showed that: intrinsic motivation was positively related to the four types of knowledge sharing tendencies; automatic response was not significantly related to creative self-efficacy; rational reflection was positively associated with creative self-efficacy; but ridiculed and deprived reflection were negatively related to creative self-efficacy. These results can be applied to encourage employees to practice rational reflection in knowledge sharing to enhance their creative self-efficacy in intelligent transportation jobs.

## Introduction

Knowledge sharing is one of the key points of knowledge management in organizations. It enables knowledge to be created, accessed, and used by others (Pangil and Nasurdin, 2019). The important process of collaborative knowledge sharing involves reducing conflict and assisting groups in meeting their shared organizational, social, and economic goals (Conley and Moote, 2003). To explain the individual cognitive process involved in knowledge sharing, Kahneman and Tversky (1979) proposed the dual-process theory (DPT) with a twofold or dual-process cognitive model (Sowden et al., 2014). In this model, Type 1 refers to fast and automatic processes, while Type 2 refers to slow and deliberate processes that involve reflective thought (Evans, 2011; Svedholm-Häkkinen, 2015). If one takes time to reflect on one’s thoughts with logic and rational analysis, then another type of mental processing will be included in one’s emotional reflection (Yang and Mattila, 2020). That is, individuals’ reflective reasoning can affect their emotion (e.g., social anxiety) and raise social conformity during social interaction (Vroling et al., 2016). Furthermore, according to the *I’m OK, You’re OK* theory (Harris, 2004), there are two factors which influence knowledge sharing: opportunistic and self-interested thinking (Estrada et al., 2016). Jiang et al. (2015) explained that people with an opportunistic tendency worry that their ideas will not be accepted or will be ridiculed during knowledge sharing. On the other hand, people with a self-interested tendency want to prevent their ideas from being stolen. Considering this, ridiculed reflection and stolen reflection can be included in the four types of knowledge sharing. Thus, extending from Kahneman’s (2011) types of system thinking, the present study identified four processes during which individuals are involved in knowledge sharing: automatic response, rational reflection, ridiculed reflection, and stolen reflection.

Workers in intelligent transportation companies are mainly responsible for planning, construction, and problem solving related to traffic management, such as implementing roadside traffic flow detection systems. In particular, highway service patrols, advanced vehicle location systems, and some elements of employee transit pass programs have embedded feedback mechanisms to measure the results of solutions relevant to the transportation industry in which each process has different problems that need to be solved creatively (Khattak et al., 2006; Iyer, 2021).

Two significant developments of social cognitive theory have integrated personal influences into Bandura’s theory. The first was self-efficacy (Bandura, 1977) and the second was reciprocal interactions. Moreover, action engagement of reciprocal interactions to develop self-efficacy is based on a distinct motivational system (Schunk and DiBenedetto, 2020). Considering that employees who work in ITI have to solve problems creatively, their work motivation may regulate their knowledge-sharing actions and creative efficacy development. Accordingly, the present study examined the links between intrinsic motivation, knowledge sharing type, and creative self-efficacy.

## Theoretical Background

### Intrinsic Motivation

“Motivation refers to processes that instigate and sustain goal-directed activities” (Schunk and DiBenedetto, 2020). Employees’ work-related motivation is expected to represent their self-imposed intentions and demands within their own work environment (Locke and Latham, 2004), and to determine the direction, intensity, and duration of an individual’s work (Van Iddekinge et al., 2018). Intrinsic motivation means that employees see work as a reward in itself, and are able to decide for themselves how they want to act and choose what they want to do to achieve their goals (Chen et al., 2018). As intrinsic motivation refers to whether workers get what they want out of their work, whether it enhances their experience, and whether they feel a sense of enjoyment and accomplishment in the process (Kanfer et al., 2013), in this study, we focus specifically on employees’ intrinsic motivation.

### Creative Self-Efficacy

Self-efficacy should be construed as a context-specific ability concept (To et al., 2020). Tierney and Farmer (2002) proposed the idea of CSE based on the study of self-efficacy and the theory of creativity, and argued that unlike other self-efficacies that include emotions such as self-esteem and self-confidence in a broad sense, CSE is more oriented toward the ability to evaluate one’s performance of creative activities. When employees are engaged in work that they are interested in and comfortable with, the more confident they are in their abilities, the more they can develop different and effective approaches, and the more active they are in solving the difficulties and dealing with the uncertain risks they encounter during the innovation process, resulting in higher creative performance (Gong et al., 2019). How CSE affects individuals’ ability or confidence they may have in their intentions to carry out a certain conduct has been well researched (e.g., Liu et al., 2016; Newman et al., 2018; Tantawy et al., 2021). In this study, we adopted a slightly different definition of CSE that is more inclined to the self-concept of ability of creative activities in ITI.

### Knowledge Sharing

Knowledge sharing is a process of transferring knowledge by any means, and generating new knowledge by interacting with others or by discussing the knowledge known to the individual with the knowledge transfer (Wijnhoven, 1998), which can make knowledge available to others in the organization (Bavik et al., 2018). Drawing on the social cognitive theory (SCT) (Bandura, 1986), collective knowledge sharing encourages the development of knowledge as employees engage in creative processes for individual work that is an essential element influencing creative behavior and outcomes (Tantawy et al., 2021). Knowledge sharing must be based on trust between employees and mutual discussion in order to spread knowledge. On the other hand, “knowledge hiding” refers to an individual’s intentional efforts to withhold or conceal knowledge requested by another (Pereira and Mohiya, 2021). Knowledge sharing actions that are ‘goal-directed’ are controlled by the desire to enhance individual ability in creative work (Sun et al., 2022). Four types of knowledge sharing were developed in this study, and the role that those knowledge sharing processes play in ITI employees’ willingness to share or hide knowledge was explored.

## Research Model and Hypotheses

### Research Model

**FIGURE 1:**
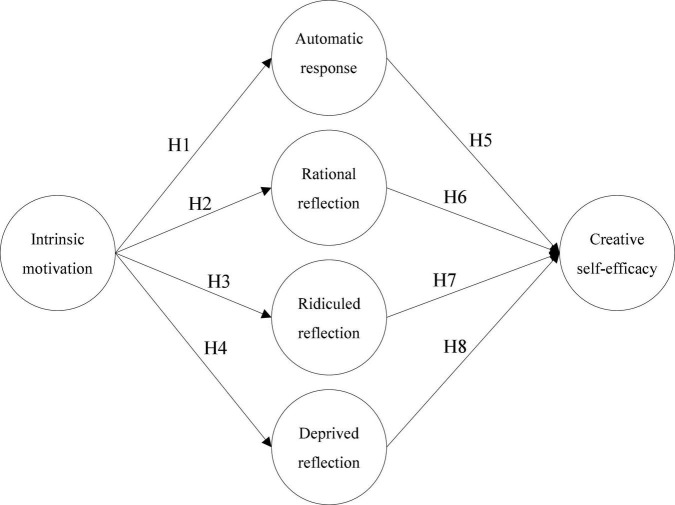
Research model.

According to the motivation-action-outcome (MAO) model (Abiero and Bradfield, 2021), the action captures procedural processes (i.e., action selection) and motivation guides how to perform an action, and is essential to the evaluation of the outcome (Sun et al., 2022). In line with this model, the present study compiled intrinsic motivation, four types of knowledge sharing in ITI, and CSE development in ITI, and proposed a research framework and research hypotheses based on the relationships among the variables as shown in Figure 1.

### Intrinsic Motivation and Knowledge Sharing

Motivational processes are those personal or internal processes that result in actions such as choice, effort, or persistence (Schunk and DiBenedetto, 2020). Self-determination theory (Ryan and Deci, 2000) conceptualizes intrinsic motivation as an autonomous form of motivation, which is represented by distinct behavioral regulations (Courtney et al., 2021). When their intrinsic motivation is high, employees are able to enjoy the process of performing their work tasks, and their behavior will be determined less by the external work environment and more by their personal behavior (Llopis and Foss, 2016). People with intrinsic motivation are more willing to actively share their knowledge, and in the process of sharing that knowledge, they will gain more recognition and satisfaction (Kao, 2012; Shen and Chang, 2018). For example, Wang and Hou (2015) drew on the self-determination theory to examine a model of the influence of motivations on employees’ knowledge sharing behaviors, and found that the correlation was positive. However, few studies have attempted to understand how employees’ intrinsic motivation predicts the four types of knowledge sharing behavior; thus, the present study proposed four hypotheses to test the predictive power as follows:

H1: Intrinsic motivation is positively related to automatic response.
H2: Intrinsic motivation is positively related to rational reflection.
H3: Intrinsic motivation is positively related to ridiculed reflection.
H4: Intrinsic motivation is positively related to deprived reflection.

### Knowledge Sharing and Creative Self-Efficacy

Yoon and Han (2018) showed that knowledge sharing is higher when employees are more innovative. Sharing knowledge can encourage employees to generate novel ideas which can then promote their individual creativity (Jyoti and Dev, 2015). Hong et al. (2004) investigated the relationship between knowledge sharing and organizational climate among R&D employees, which was guided by primary relationship norms to explore the knowledge sharing attitudes, and found that cognitive environmental evaluation could drive creative self-efficacy. For example, knowledge sharing behaviors in online knowledge communities that guides intellectual stimulation for employees in which their novel thinking can be triggered, thus leading to innovative solutions (Tantawy et al., 2021). Based on the above, this study aimed to understand whether ITI employees with the four types of higher knowledge sharing behavior had higher CSE. We therefore proposed the following hypotheses:

H5: Automatic response is positively related to CSE.
H6: Rational reflection is positively related to CSE.
H7: Ridiculed reflection is positively related to CSE.
H8: Deprived reflection is positively related to CSE.

### Intrinsic Motivation and Creative Self-Efficacy

Liu and Zhang (2007) showed that employees’ internal motivation is a significant factor in their innovative performance and has a positive effect on both idea generation and execution. As creativity requires a higher degree of IM, it is important to encourage employees to work hard in order to attain breakthroughs (Laforet, 2011). By sharing knowledge, employees with a high level of intrinsic work motivation can be stimulated to be creative in successfully completing their assignments, and new intellectual pathways can also be generated to help them experience higher creativity (Shafi et al., 2020). CSE is related to the ability to produce creative output, and can reflect intrinsic motivation to engage in creative activities (Tantawy et al., 2021). Thus, the following hypothesis that explores the mediated role of the four types of knowledge sharing between intrinsic motivation and CSE in ITI was proposed:

H9: Intrinsic motivation is positively related to CSE mediated by knowledge sharing.

## Research Design

### Procedure

Intelligent transportation jobs include the construction of software and hardware equipment in the traffic control center and various types of roadside equipment. Intelligent transportation implements cloud-based IoT based on the availability of accurate and timely information for the processes of forecasting and planning, resources, logistics service management as well as the many sub-processes in the supply chain (Jiang et al., 2020). The most important goal is to improve the accuracy rate of each type of equipment beyond the requirements of the user unit, and at the same time, to integrate all the various types of equipment installed on the roadside and to share the real-time information with users through the integrated system (Boukerche et al., 2020). Therefore, all tasks require the participation of experienced colleagues, as well as the cooperation of new colleagues and downstream vendors. Each person needs to share knowledge to solve internal and external problems to work out projects, given employees’ uneven experience and individual professional differences.

This study was conducted to investigate the relationship between intelligent transportation employees’ intrinsic motivation and their knowledge sharing CSE by using currently working employees as the empirical subjects. Adopting snowball sampling, a message was sent to the target key personnel in the human resource departments of intelligent transportation companies asking them to deliver the questionnaire link to their colleagues. A message was also embedded in the questionnaire that asked participants to pass on the questionnaire link to friends who worked in technology-related jobs. The survey was conducted from January to February 2021.

Regarding ethical considerations, a message appeared in the introduction section of the questionnaire indicating the purpose of the survey and informing participants that they were free to refuse to reply, and that the collected data would only be used for this study.

### Participants

A total of 262 questionnaires were collected, and 18 invalid questionnaires were deleted. This gave 232 valid questionnaires for use in the further statistical analysis. The distribution of the sample in this study is analyzed as follows: in terms of gender, 106 (45.7%) respondents were male and 126 (54.3%) were female; regarding age, 29 (12.5%) were 25 years old or below, 48 (20.7%) were 26 ∼ 30 years old, 41 (17.7%) were 31 ∼ 35 years old, 31 (13.4%) were 36 ∼ 40 years old, and 83 (26.7%) were 41 years old or above; as for completed educational level, 73 (40.1%) had graduated from junior college, 105 (45.3%) had graduated from university, and 34 (14.7%) had graduate degrees.

### Questionnaire

We referred to the relevant literature to develop the questionnaire items on intrinsic motivation, CSE, and knowledge sharing. In this section, we discuss the composition, measurement, and operational definitions of each variable. To ensure content validity, five domain experts were invited to check the accuracy and applicability of the item translation; to ensure face validity, 10 students were invited to respond to the items to check if any statements needed revising. The questionnaire used a 5-point Likert scale, with 1 for *strongly disagree*, 2 for *disagree*, 3 for *neutral*, 4 for *agree*, and 5 for *strongly agree*. The reliability and validity of the items and constructs were re-tested according to the confirmatory study (see Tables 2, 3).

#### Intrinsic Motivation

This scale was modified from the intrinsic motivation part of the job preference scale proposed by Amabile et al. (1994), and is mainly used to measure the intrinsic motivation of employees regarding their jobs. An example item is: I hope my job provides me with opportunities to increase my knowledge and skills.

#### Knowledge Sharing

This study modified the knowledge sharing scale proposed by Hong et al. (2004) and Hong et al. (2020) by applying the good trust theory and the two-system theory. An example item of automatic response is: When I think of knowledge to share, I immediately express my opinion. An example item of rational reflection is: When I think of knowledge to share, I explain the reasonableness of the idea in my mind before I express my opinion. An example item of ridiculed reflection is: When I think of knowledge to share, I will first think whether it will be rejected before I express my opinion. An example item of deprived reflection is: When I think of an idea or thought, I think about it first and see if it will be used by others in other topics before expressing my opinion.

#### Creative Self-Efficacy

This study used Carmeli and Schaubroeck’s (2007) Creativity Efficacy Scale, which integrates the concepts of self-efficacy and creativity. The content of the scale includes self-assessment of employees’ creativity performance, whether they feel confident in accomplishing their work goals creatively, and whether they feel capable of solving problems creatively. An example item is: I am confident that I have the ability to use my creativity to solve problems.

## Results

### Item Analysis

The purpose of the first-order validation factor analysis was to remove non-identifiable items. First, the original items of each construct were subjected to first-order validation analysis to examine the relationship between each item in each construct. Second, the fitness was checked to see if it was within the criteria, which according to Hair et al. (2019) included a χ^2^/df ratio of less than 5, a GFI greater than 0.80, an AGFI greater than 0.80, and an RMSEA less than 0.10, as shown in Table 1.

**TABLE 1:** Item analysis.

Index	χ^2^/df	RMSEA	GFI	AGFI	*t*
Threshold	<5	<0.10	>0.80	>0.80	>3
Intrinsic motivation	2.4	0.08	0.99	0.95	12.41 ∼ 15.95
Automatic response	2.2	0.07	0.99	0.95	18.26 ∼ 20.12
Rational reflection	1.15	0.02	0.99	0.98	11.37 ∼ 17.00
Ridiculed reflection	0.05	0.01	0.99	0.99	15.07 ∼ 15.72
Deprived reflection	2.85	0.09	0.99	0.94	20.21 ∼ 22.29
CSE	2.8	0.09	0.98	0.94	13.41 ∼ 16.69

**TABLE 2:** Analysis of validity and reliability.

Construct	*M*	*SD*	α	CR	AVE	FL
Threshold	–	–	>0.70	>0.70	>0.50	>0.50
Intrinsic motivation	3.37	0.71	0.85	0.85	0.59	0.77
Automatic response	3.51	0.70	0.89	0.89	0.67	0.82
Rational reflection	3.75	0.66	0.88	0.88	0.65	0.80
Ridiculed reflection	3.49	0.85	0.93	0.93	0.77	0.83
Deprived reflection	3.15	0.92	0.93	0.93	0.77	0.87
CSE	3.52	0.76	0.94	0.93	0.74	0.86

**TABLE 3:** Discrimination validity analysis.

Construct	1	2	3	4	5	6
(1) Intrinsic motivation	0.77					
(2) Automatic response	0.41	0.82				
(3) Rational reflection	0.36	0.48	0.81			
(4) Ridiculed reflection	0.29	0.07	0.03	0.88		
(5) Deprived reflection	0.35	0.23	0.09	0.42	0.88	
(6) CSE	0.38	0.23	0.40	–0.21	–0.17	0.86

### Analysis of Validity and Reliability

In this study, Cronbach’s α and composite reliability (CR) were used to examine the reliability analysis of the constructs and the overall questionnaire, and SPSS 23 statistical analysis software was used to perform the analysis. The higher the value, the higher the reliability consistency of the scale. To enable a more accurate measurement, this study also relied on the Cronbach’s α and CR value of the structural equation model to analyze the data, where good values are usually above 0.70 (Cook and Beckman, 2006). The Cronbach’s α values of the constructs ranged from 0.85 to 0.94, and the CR values of the constructs ranged from 0.85 to 0.93, as shown in Table 2.

According to Fornell and Larcker’s (1981) criteria for assessing convergent validity, a higher convergent validity represents a high FL value, which means that the tested variables can be converted to a potential variable, and generally an acceptable FL value should be at least 0.50. The FL values of the constructs ranged from 0.77 to 0.87, as shown in Table 2.

Cook and Beckman (2006) stated that the average variance extracted (AVE) value needs to be more than 0.50 to represent the effect of averaging on a construct. This value is the sum of the squares of the standardized factor loadings of the constructs, and if this criterion is reached, it means that each question can be explained by each construct. The AVE values of the constructs ranged from 0.59 to 0.77, as shown in Table 2.

The average variance extracted for each construct must be greater than the squared correlation coefficient between the two compared constructs in order to represent the discriminant validity of each construct (Fornell and Larcker, 1981). As shown in Table 3, the square root of the AVE for each construct ranged from 0.77 to 0.86, all of which were greater than the correlation coefficients between constructs.

### Model Fit Analysis

The study model was finally analyzed for overall fitness using the AMOS 20.0 statistical software (Hair et al., 2019). RMSEA = 0.05, GFI = 0.88, AGFI = 0.85, NFI = 0.91, and NNFI = 0.96, where greater than 0.90 meets the criteria. CFI = 0.97, IFI = 0.97, and RFI = 0.90, where greater than 0.80 meets the criteria. PNFI = 0.81 and PGFI = 0.72, where greater than 0.50 meets the criteria.

### Path Analysis

**FIGURE 2:**
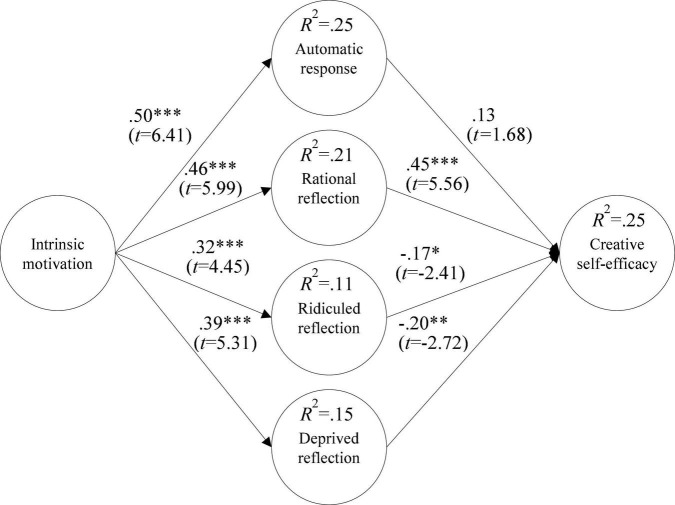
Research model verification. *p < 0.05, ^**^p < 0.01, ^***^p < 0.001.

Intrinsic motivation and automatic response have a positive relation (β = 0.50^***^, *p* < 0.001); intrinsic motivation and rational reflection have a positive relation (β = 0.46^***^, *p* < 0.001); intrinsic motivation and ridiculed reflection have a positive relation (β = 0.32^***^, *p* < 0.001); intrinsic motivation has a positive relation with deprived reflection (β = 0.39^***^, *p* < 0.001); automatic response has no relation with CSE (β = 0.13, *p* > 0.05); rational reflection has a positive relation with CSE (β = 0.45^***^, *p* < 0.001); ridiculed reflection has a negative relation with CSE (β = −0.17*, *p* < 0.05); and deprived reflection has a negative relation with CSE (β = −0.20^**^, *p* < 0.01), as shown in Figure 2.

The explanatory power of intrinsic motivation for automatic response is 25%; the explanatory power of intrinsic motivation for rational reflection is 21%; the explanatory power of intrinsic motivation for ridiculed reflection is 11%; and the explanatory power of intrinsic motivation for deprived reflection is 15%. Automatic response, rational reflection, ridiculed reflection, and deprived reflection all have explanatory power of 25% for CSE, as shown in Figure 2.

### Indirect Effects Analysis

In terms of indirect effects, intrinsic motivation had an indirect positive relation with CSE (β = 0.14*, *p* < 0.05), as shown in Table 4.

**TABLE 4:** Indirect effect analysis.

Construct	Intrinsic motivation	
	β	95% CI
CSE	0.14*	[0.01, 0.28]

*p < 0.05.

## Discussion

The results of this study showed that the intrinsic motivation of employees in intelligent transportation companies was positively related to the four types of knowledge sharing. Intrinsic motivation is also considered to be an important factor influencing knowledge sharing behavior because such positive beliefs motivate group members to cooperate and share knowledge with each other for maximum benefit (Tseng and Kuo, 2010). In addition, Liu and Zhang (2007) argued that employees’ internal motivation is an important factor in their innovation performance and has a positive impact on both idea generation and execution, whereas external motivation only has an impact on execution and not on idea generation at all. Moreover, when internal motivation is high, employees are able to enjoy the process of performing their tasks (Llopis and Foss, 2016). Kao (2012) and Shen and Chang (2018) suggested that people with intrinsic motivation are more motivated and pursue self-actualization, and therefore are more willing to actively share their knowledge. This finding is not contrary to common sense, because knowledge sharing is usually a voluntary behavior. Therefore, employees usually do not share valuable knowledge or actively participate or cooperate without any intrinsic motivation (Nguyen et al., 2019). The results of this study are consistent with the findings of previous scholarly studies, and with the research hypotheses of this study.

Collective creativity is regulated by a series of interactions involving knowledge sharing, resulting in new ideas, approaches, and discoveries (Parjanen and Hyypiä, 2019). Knowledge sharing contributes to reflection practice to enhance creativity development (Tang et al., 2020). After a period of reflection, participants would generate ideas with higher originality, which is then related to improvement in performance (Hao et al., 2016). It can be assumed that participants should practice knowledge sharing, so the four types of knowledge sharing facilitating CSE were elaborated in this study.

Sharing knowledge to enhance collective creativity has not always been implemented effectively in industry (Parjanen and Hyypiä, 2019). A previous study revealed that when employees with a higher sense of self-efficacy have a higher sense of responsibility for knowledge sharing, they will share their knowledge more spontaneously (Liu and Liu, 2011). On the other hand, Tantawy et al. (2021) argued that by stimulating employees’ intelligence via knowledge sharing, new ideas can be generated to achieve innovative solutions. Jyoti and Dev (2015) found that shared knowledge can encourage employees to generate new ideas to enhance their personal creativity. Yoon and Han (2018) stated that knowledge sharing is more common when employees are more innovative, and they will have higher levels of self-efficacy. However, this study found that not all types of knowledge sharing among employees in ITI are helpful for CSE. That is, it is a fundamental requirement that employees working on jobs need innovation, but if they have automatic responses without reflecting enough on the condition, then the knowledge they share cannot be innovative (Calavia et al., 2021). The results of this study showed that automatic response had no relation with CSE, whereas ridiculed and stolen reflection were negatively related to CSE. However, rational reflection was positively associated with CSE.

Sylvie (2011) suggests that creativity requires a higher level of intrinsic motivation and therefore employees should be encouraged to work hard to achieve higher levels of creative work outcomes. Shafi et al. (2020) argued that through knowledge sharing, employees with high levels of intrinsic motivation can be motivated to be creative in successfully completing tasks, and can stimulate new intellectual pathways to generate higher levels of creativity. Therefore, Jyoti and Dev (2015) emphasized the need to examine the moderating variables in order to form strong relationships and knowledge sharing results. This study investigated the mediating effects of knowledge sharing types on intrinsic motivation and CSE, and the results of the analysis of this study confirmed the role of the four types of knowledge sharing in the mediation between intrinsic motivation and CSE. The results of this study confirmed the mediating effect of the four types of knowledge sharing in ITI between intrinsic motivation and CSE.

## Conclusion

In recent years, attention has been paid to intelligent transportation systems because of the growing demands for road safety and efficiency in today’s highly interconnected road networks. We recruited ITI practitioners as research participants to expand our understanding of the organizational behavior of this industry. In this study, knowledge sharing was divided into four types that extended Kahneman’s types of systematic thinking to: automatic response, rational reflection, ridiculed reflection, and deprived reflection.

From the study results, it was found that the higher the rational reflection in knowledge sharing that employees in ITI have, the higher their level of CSE. Additionally, the more intrinsic motivation employees have, the more knowledge sharing actions they are likely to engage in as part of their creative work. In addition, the results of this study revealed that not all types of knowledge sharing are beneficial to CSE. Organizations should pay special attention to avoid the generation of automatic responses, ridiculed reflection, and deprived reflection when recruiting or conducting employee training. It is also important to enhance employees’ rational reflection in knowledge sharing to promote CSE for solving ITI-related problems.

From the results of the study as well as from the previous literature, motivation has always been an important issue in organizational behavior, and when employees are motivated by their own preferences and ideas to get their work done, they perform better than those who do not work according to their own preferences. Therefore, in terms of candidates, we can look for employees with strong intrinsic motivation, so that they can devote themselves to their work and actively engage in knowledge sharing with other employees. Therefore, in order to increase knowledge sharing among ITI employees, managers may adopt some approaches to motivate them to share knowledge.

### Research Limitations and Future Study

Each industry has its own characteristics, and each company also has a different culture. As people are usually influenced by the herd effect, when it is discovered that most people in a group perform a particular behavior, there will be an invisible pressure that drives others to follow the behavior of the majority. Therefore, it is recommended that subsequent researchers can conduct research on different industries and conduct further discussions.

As technology advances and helps virtual communities and online platforms flourish, this has led to increasingly diversified ways of knowledge sharing, and a growing number of people paying attention to the effectiveness of different knowledge sharing systems. Therefore, in the future, we can also explore the impact of different ways of knowledge sharing on employees’ CSE or work performance.

## Data Availability Statement

The original contributions presented in the study are included in the article/supplementary material, further inquiries can be directed to the corresponding authors.

## Ethics Statement

Ethical review and approval was not required for the study on human participants in accordance with the local legislation and institutional requirements. The patients/participants provided their written informed consent to participate in this study.

## Author Contributions

J-CH, YS, and J-HY: concept and design and drafting of the manuscript. J-HY and YS: acquisition of data and statistical analysis. J-CH and YS: critical revision of the manuscript. All authors contributed to the article and approved the submitted version.

## Conflict of Interest

The authors declare that the research was conducted in the absence of any commercial or financial relationships that could be construed as a potential conflict of interest.

## Publisher’s Note

All claims expressed in this article are solely those of the authors and do not necessarily represent those of their affiliated organizations, or those of the publisher, the editors and the reviewers. Any product that may be evaluated in this article, or claim that may be made by its manufacturer, is not guaranteed or endorsed by the publisher.
